# The effects of psychological treatments of depression in children and adolescents on response, reliable change, and deterioration: a systematic review and meta-analysis

**DOI:** 10.1007/s00787-021-01884-6

**Published:** 2021-10-06

**Authors:** Pim Cuijpers, Eirini Karyotaki, Marketa Ciharova, Clara Miguel, Hisashi Noma, Yvonne Stikkelbroek, John R. Weisz, Toshi A. Furukawa

**Affiliations:** 1 Department of Clinical, Neuro and Developmental Psychology, Amsterdam Public Health research institute, Vrije Universiteit, Van der Boechorststraat 7-9, 1081 BT Amsterdam, The Netherlands; 2 WHO Collaborating Centre for Research and Dissemination of Psychological Interventions, Vrije Universiteit Amsterdam, Amsterdam,, The Netherlands; 3 Department of Data Science, Institute of Statistical Mathematics, Tokyo, Japan; 4 Department of Clinical Child and Family Studies, Faculty of Social and Behavioral Sciences, Utrecht University, Utrecht, The Netherlands; 5 GGZ Oost Brabant, P.O. Box 3, 5427 ZG Boekel, The Netherlands; 6 Harvard University, Cambridge, Massachusetts USA; 7 Department of Health Promotion and Human Behavior, Kyoto University Graduate School of Medicine/School of Public Health, Kyoto, Japan

**Keywords:** Major depressive disorder, Depression, Meta-analysis, Psychotherapy, Cognitive behaviour therapy

## Abstract

Meta-analyses show that psychotherapies are effective in the treatment of depression in children and adolescents. However, these effects are usually reported in terms of effect sizes. For patients and clinicians, it is important to know whether patients achieve a clinically significant improvement or deterioration. We conducted such a meta-analysis to examine response, clinically significant change, clinically significant deterioration and recovery as outcomes. We searched four bibliographic databases and included 40 randomised trials comparing psychotherapy for youth depression against control conditions. We used a validated method to estimate outcome rates, based on means, standard deviation and N at baseline and post-test. We also calculated numbers-need-to- treat (NNT). The overall response rate in psychotherapies at 2 (±1) months after baseline was 39% (95% CI: 34–45) and 24% (95% CI: 0.19–28) in control conditions (NNT: 6.2). The difference between therapy and control was still significant at 6–12 months after baseline (NNT=7.8). Clinically significant improvement was found in 54% of youth in therapy, compared with 32% in control groups (NNT=5.3); clinically significant deterioration was 6% in therapy, 13% in controls (NNT=5.1); recovery was 58% in therapy, 36% in controls (NNT=3.3). Smaller effects were found in studies with low risk of bias. Psychotherapies for depression in youth are effective compared to control conditions, but more than 60% of youth receiving therapy do not respond. More effective treatments and treatment strategies are clearly needed. *Trial registration* https://osf.io/84xka

## Introduction

It has been estimated that almost 14% of all adolescents will meet criteria for a depressive disorder before the age of 18 [1]. Depression in children and adolescents does not only lead to personal suffering in those affected and their families, but it is also associated with increased suicide risk [2] and functional impairment at home, school and society [3, 4]. Several important negative health outcomes in adulthood have been associated with depression in children and adolescents, including poorer self-perceived general health, higher health care utilization and increased work impairment due to physical health [5]. With an estimated prevalence of 2.6% [6] and a much higher and increasing prevalence rate during adolescence, depression is undoubtedly a major public health challenge.

Psychological treatments are considered to be one of the main treatment options for youth depression and meta-analyses have shown that these treatments are indeed effective, although the effects are modest, [7] and smaller than those in adults, especially in younger children [8]. Although most studies have focused on cognitive behaviour therapy (CBT) and to a smaller extent interpersonal therapy (IPT), several other types of therapy have also been examined in randomised controlled trials, including behavioural activation [9], problem-solving therapy [10] and family therapy [11].

Most meta-analyses, however, report the effects of psychotherapies in terms of standardized mean differences (SMD), such as Cohens’ d and Hedges’ g, indicating the difference between the therapy and a control group after the treatment in terms of standard deviation. For patients, their families and clinicians, however, clinical significant change is much more important, because it indicates the chance of getting better after a treatment, and to compare that with the chance of getting better without treatment. The SMD is not very informative in this respect and cannot be seen as an indicator of clinical relevance, because it is still a statistical concept [12, 13].

Binary outcomes, such as response or remission are easier to understand, because they indicate how many patients get substantially better after treatment. Such outcomes are often presented as Relative Risks (RRs) or Odds Ratios (ORs) and indicate the relative benefit of a treatment in comparison to a control condition or another treatment. This is easier to interpret than effect sizes, but these outcomes still do not indicate the chance of getting better when receiving treatment [13].

Simply knowing the chance of getting better with or without a treatment is the most informative outcome for many patients and their families as well as for clinicians. Meta-analyses rarely report these outcomes, however, because heterogeneity is typically very high when proportions are pooled. Nevertheless, the clinical relevance of these outcomes is so high, that we believe that pooling them is still important. Pooling of binary data is also done in other important areas where high levels of heterogeneity are found, such as meta-analyses of prevalence rates [14–16].

Unfortunately, most randomised trials examining psychotherapy for depression in youth, usually do not report binary outcomes, but only means and standard deviation of the treatment and control groups. However, there is a well-validated method to estimate binary outcomes in psychotherapy and control conditions using estimates based on the means at baseline, and the means, standard deviations and N at post-test [17]. This method estimates how many patients are scoring above or below a cut-off assuming a normal distribution of the outcome. For example, the cut-off value for response (50% reduction of depressive symptoms from baseline to post-test) can be estimated from the baseline means, by simply taking 50% of the score at baseline. Then, it can be estimated with the means, standard deviation and N at post-test how many participants reached this cut-off value for response, assuming a normal distribution of the outcome measure. This method can also be used to estimate other binary outcomes, as long as they can be estimated with the baseline and post-test measures. In a previous meta-analysis, we found a correlation of 0.94 between the response and remission rates reported in the paper and the estimated rates using this method [18]. This method not only allows to estimate binary outcomes, but also to calculate numbers-needed-to-treat (NNTs), indicating how many patients have to be treated to have one more positive outcome compared to the comparison group [19].

This method also allows to calculate negative effects of psychotherapies. It is now broadly acknowledged that negative effects are a core issue in research and practice of psychological intervention in general [20]. Although it has long been assumed that no harm can be done, because psychotherapy is “only talking”, much research has by now shown that some patients do deteriorate during therapy [20]. To the best of our knowledge, however, negative effects in psychotherapies for depression in youth have hardly been examined. The method described above to estimate binary outcomes based on means at baseline and post-test, and the N and standard deviation at post-test, can also be used to estimate clinically significant deterioration and get a first rough estimate of negative effects of these therapies.

We decided to conduct a meta-analysis of psychological treatments of depression in children and adolescents, aimed at examining binary outcomes using the validated method to estimate these outcomes.

## Methods

### Search strategy and selection criteria

The protocol for this meta-analysis was registered at the Open Science Framework (https://osf.io/84xka) [21]. We used an existing database of randomised trials on the psychological treatment of depression, which includes trials in adults and in children and adolescents [22]. The database is continuously updated and was developed through a comprehensive literature search (up to Jan 1st, 2021). For this database, we searched four major bibliographical databases (PubMed, PsycINFO, Embase, Cochrane Library) by combining index and free terms indicative of depression and psychotherapies, with filters for randomized controlled trials. The full search string for PubMed is available in Supplement 1 and all search strings can be found at the project’s website (www.metapsy.org). Trials in children and adolescents were also identified through a recent other meta-analysis of psychotherapies in youth [7, 23]. All records were screened by two independent researchers, and all papers that could possibly meet inclusion criteria according to one of the researchers were retrieved as full text. The decision to include or exclude a study in the database was also done by two independent researchers, and any disagreements were solved through discussion and consensus.

For the current meta-analysis, we included (a) randomized trials (b) in which a psychological treatment (c) for depression in children and adolescents (d) was compared with a control group (waitlist, CAU, other control). We included studies in which the presence of a depressive disorder was established using a diagnostic interview as well as studies in which participants had to score above a cut-off on a self-report depression scale. Studies which included both adolescents and adults were excluded from this meta-analysis. No language restrictions were applied.

### Quality assessment and data extraction

We assessed the validity of included studies using four criteria of the Cochrane ‘Risk of bias assessment tool [24]: allocation sequence generation; concealment of allocation to conditions; prevention of knowledge of the allocated intervention (masking of assessors); and dealing with incomplete outcome data (this was assessed as low risk when intention-to-treat analyses were conducted). Items were dichotomized as low or high/unclear risk. These assessments were conducted by two independent researchers, and disagreements were solved through discussion.

We also coded participant characteristics, study characteristics, and the time from baseline to outcome.

### Outcome measures

Treatment response (50% reduction in depressive symptomatology between baseline and post-test) was the primary outcome [18]. We retrieved all response rates at all time points that were reported in the included studies, but we focused the main analyses on response rates at 2 (± 1) months after baseline, because this was the post-test for most studies and most interventions ended at that time. We clustered the studies according to the time from baseline to post-test, because absolute rates of outcomes are also influenced by spontaneous recovery rates and pooling different times of outcome would introduce considerable heterogeneity. When more than one outcome measure was reported, we selected the outcome according to an algorithm that has been used in previous meta-analyses (meaning that when more than one outcome measure was used, we selected the outcomes with priority for: Hamilton Depression Rating Scale (HAM-D), Beck Depression Inventory I or II (BDI), Children's Depression Inventory (CDI), and the revised Reynolds Adolescent Depression Scale (RADS-R) [8]. If the response rate was not reported in the paper, we estimated it with the well-validated method using estimates based on the means at baseline, and the means, standard deviations and N at post-test [17]. If neither the response rate, nor the data to estimate it were reported, the study was excluded.

The main outcome was the response rate at post-test, assuming that all study drop-outs were non-responders, because this was considered to be the most conservative estimate. We also conducted two sensitivity analyses in which: (a) all participants lost to follow-up were considered as responders, and (b) only study completers were included. We categorized the response rates according to the time between baseline and post-test and selected the post-test at 2 (±1) months after baseline as the main outcome, but also calculated response rates at later follow-up times.

We also calculated the Reliable Change Index, which is a psychometric criterion used to evaluate whether the change between baseline and post-test is considered statistically significant (the difference between baseline and post-test means divided by the standard error of the difference between the two scores is greater than 1.96, conservatively assuming a Cronbach’s alpha of 0.75) [25]. We used the same method to calculate the Reliable Deterioration Index, indicating whether a patient reliably deteriorated, as an indicator of negative effects. Because there were several studies in which all participants met criteria for a depressive disorder at baseline according to a diagnostic interview, we also calculated recovery (the proportion of participants not meeting criteria for a disorder at post-test anymore).

### Meta-analyses

We first pooled rates for response, reliable change, reliable deterioration and recovery using the “metaprop” command of the “meta” package in R (version 3.6.3). In these analyses, we synthesized the binomial outcome data by random-effects pooling models after transforming to a logit scale. The pooled summary results were converted to the raw proportion scale, and the estimates and their 95% confidence intervals (CIs) are presented. Because we expected considerable heterogeneity, we employed a random effects pooling model in all analyses, according to the DerSimonian-Laird method. As indicator of heterogeneity, we calculated the *I*^*2*^ statistic and its 95% CI [26]. In addition, we calculated the prediction interval, which indicates the range in which the true effect size of 95% of future studies will fall.

First, we meta-analysed response rates for psychotherapies and control conditions separately at 2 (±1 month) follow-up (our primary outcome). We also pooled response rates assuming that all dropouts are responders, as well as the rates for the completers of the study. To improve the interpretation of the results, we also generated an l’Abbé plot with the response rate in the control group at the horizontal axis and the response rates in the treatment group at the vertical axis [27]. The 45° line indicates no effect.

We then examined the risk of small study effects by testing asymmetry through Egger’s test and adjusted the rates for the small study effects through Duval and Tweedie trim-and-fill procedure (R0 estimator) [28]. We also conducted sensitivity analyses by excluding outliers, defined as studies whose 95% CI of the response rate does not overlap with the 95% CI of the response rate of the pooled studies, by limiting the analyses to those studies with low risk of bias, and by limiting the analyses to those studies that reported response rates in the papers.

In the next step, we meta-analysed the Relative Risk (RR) of response. Then, we calculated the NNT using the pooled RR and the response rate in the control group, as recommended by the Cochrane Collaboration [29].

To examine potential sources of heterogeneity, we conducted subgroup analyses with age category (adolescents; children), recruitment (only clinical samples; other recruitment), diagnosis (diagnosed depressive disorder; subthreshold depression; scoring above a cut-off); type of psychotherapy (CBT; IPT; other), format (individual; group; guided self-help), risk of bias (low; other), control condition (waiting list; usual care; other). These subgroup analyses were conducted separately for the response rates in the psychotherapy conditions, the response rates in the control condition and the RRs.

We conducted the analyses for the pooled response rates at different follow-up times, as well as for reliable change and reliable deterioration.

## Results

### Selection and inclusion of studies

**Fig. 1:**
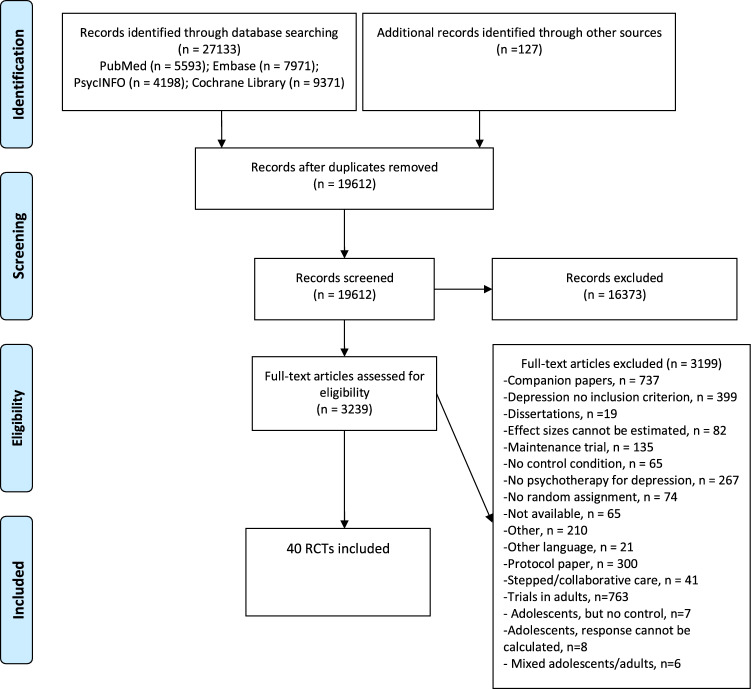
PRISMA flowchart

After examining 27,133 records (19,612 after removal of duplicates), we retrieved 3239 full-text papers for further consideration and excluded 3199 of these. The PRISMA flowchart, including the reasons for exclusion, is presented in Fig. 1. Forty studies including 3779 participants (2029 in the treatment groups and 1750 in the control groups) met our inclusion criteria. References of the included studies are given in Appendix [1].

### Characteristics of included studies

Key characteristics of the included studies are presented in Table 1. Eleven studies were aimed at children up to 12 years of age, while 29 studies were aimed at adolescents (between 12 and 18 years). In 10 studies, all participants were recruited from clinical samples, while the other 30 studies recruited participants through the community or mixed sources. Seventeen studies were aimed at youth with a diagnosed depressive disorder, 16 used a cut-off score on a self-rating depression scale to include participants, and 7 were aimed at youth with a subthreshold depression (depressive symptoms, but not meeting criteria for a diagnosed depressive disorder). The proportion of girls ranged from 32 to 100% (median 60%). The 40 studies included 46 psychotherapy conditions that were compared with a control condition (6 studies included 2 psychotherapy arms). A total of 31 of the 46 psychotherapies were CBT, 6 were IPT and 9 were characterised as another type of therapy (including problem-solving therapy, behavioural activation, family therapy, supportive therapy, among others). Eleven therapies used an individual format, 26 used a group format, 3 a guided self-help format and the other 6 used a mixed format. The number of sessions ranged from 4 to 41 with a median of 12. The control condition used in the 40 studies included waitlist control groups (12 studies), usual care (16 studies) or other control conditions (12 studies). Twenty-five studies were conducted in the United States, 9 in Europe (including the UK), and 6 in other countries. The response rates were reported in only two studies, in the other studies the response rates were estimated with the method of Furukawa and colleagues [17].

**Table 1:** Selected characteristics of randomised trials on psychotherapies for children and adolescents

Study	A/C	Clin	Diag	M_age_	Prop wom	Type	Frm	*N*_se_	Ctr	Outcome	FU	Cou	sg	ac	ba	itt	rob
Ackerson 1998	A	–	CO	16.0	0.64	cbt	oth	4	wl	HAMD	2 (±1)	US	–	–	–	–	0
Bolton 2007	A	–	CO	15.0	0.57	ipt	grp	16	oth	depr scale	4–6	Oth	+	–	+	+	3
Charkandeh 2016	A	+	DD	15.3	0.54	cbt	ind	24	wl	CDI	2 (±1)	Oth	+	–	sr	–	2
Clarke 1995	A	–	SD	15.3	0.61	cbt	grp	15	cau	HAMD	2 (±1)	US	–	–	–	–	0
Clarke 1999-cwd	A	–	DD	16.2	0.71	cbt	grp	16	wl	HAMD	2 (±1)	US	–	–	+	–	1
Clarke 1999-cwd+p						cbt	grp	16		HAMD							
Clarke 2001	A	–	SD	14.6	0.64	cbt	grp	15	cau	HAMD	2 (±1)	US	+	+	+	–	3
Clarke 2002	A	–	DD	15.3	0.69	cbt	grp	16	cau	HAMD	2 (±1)	US	–	–	+	+	2
De Cuyper 2004	C	–	SD	10.0	0.75	cbt	grp	16	wl	CDI	4-6	EU	–	–	sr	–	1
De Jonge-Heesen 2020	A	–	CO	13.6	0.64	cbt	grp	8	oth	CDI-A	2 (±1)	EU	+	+	sr	+	4
Diamond 2002	A	–	DD	14.9	0.78	oth	ind	8	wl	BDI	2 (±1)	US	–	–	+	–	1
Esposito-Smythers 2019	A	+	DD	14.9	0.76	cbt	Oth	41	cau	CDI-II	4-6	US	+	+	sr	+	4
Gillham 2006	C	–	SD	11.5	0.53	cbt	grp	12	cau	CDI	4-6	US	+	–	–	+	2
Idsoe 2019	A	–	CO	16.7	0.88	cbt	grp	8	cau	CESD	4-6	EU	–	+	sr	+	3
Israel 2013	A	+	DD	15.6	0.55	oth	ind	11	cau	HAMD	2 (±1)	EU	+	+	+	+	4
Kahn. 1990-cbt	C	–	DD	12.1	0.51	cbt	grp	12	wl	CDI	2 (±1)	US	–	–	–	+	1
Kahn 1990-other						oth	ind	12	wl	CDI							
Kitchen 2020	A	+	DD	15.7	0.82	bat	ind	8	cau	MFQ-C	2 (±1)	UK	+	+	sr	–	3
Lewinsohn 1990-cwd	A	–	DD	16.2	0.61	cbt	grp	14	wl	BDI	2 (±1)	US	–	–	sr	–	1
Lewinsohn 1990-cwd/p						cbt	grp	14	wl	BDI							
Liddle 1990	C	–	CO	9.2	0.32	cbt	grp	8	wl	CDI	2 (±1)	Oth	–	–	sr	+	2
Listug-Lunde 2013	C	–	CO	12.4	0.38	cbt	grp	13	cau	CDI	2 (±1)	US	–	–	sr	–	1
Luby 2012	C	–	DD	4.3	0.37	oth	ind	14	oth	BDI-II	2 (±1)	US	+	–	+	+	3
Martinovic 2006	A	+	SD	17.4	0.68	cbt	grp	6	cau	HAMD	4-6	EU	+	–	+	+	3
McCarty 2013	C	–	CO	12.7	0.60	cbt	grp	12	oth	MFQ C	4-6	US	+	+	+	+	4
Moeini 2019	A	–	CO	16.4	1.00	oth	Oth	8	cau	CESD	2 (±1)	Oth	–	–	sr	+	2
Mufson 1999	A	+	DD	15.8	0.73	ipt	ind	12	oth	HAMD	2 (±1)	US	+	–	+	+	3
Mufson 2004	A	+	DD	15.1	0.84	ipt	ind	12	cau	HAMD	4-6	US	+	–	+	+	3
Reynolds 1986	A	–	CO	15.7	0.63	cbt	grp	10	wl	BDI	2 (±1)	US	–	–	–	–	0
Rohde 2004	A	–	DD	15.1	0.48	cbt	grp	16	oth	HAMD	2 (±1)	US	+	–	+	+	3
Rosello 1999-CBT	A	–	DD	14.7	0.54	cbt	Oth	12	wl	CDI	2 (±1)	US	–	–	+	+	2
Rosello 1999-IPT						ipt	Oth	12	wl	CDI							
Santomauro 2016	A	–	CO	15.7	0.40	cbt	grp	11	wl	DASS	2 (±1)	Oth	+	–	sr	+	3
Stark 1987-pst	C	–	CO	11.2	0.43	oth	grp	12	wl	CDRS-R	2 (±1)	US	–	–	+	–	1
Stark 1987-selfcontrol						pst	grp	12	wl	CDRS-R							
Stice 2008-cbt	A	–	SD	15.6	0.56	cbt	grp	4	cau	CDI	2 (±1)	US	+	+	+	+	4
Stice 2008-supp-expr						sup	grp	4	cau	CDI							
Szigethy 2007	A	–	SD	15.0	0.51	cbt	ind	10	cau	CDI-CP	2 (±1)	US	–	–	+	+	2
TADS 2004	A	–	DD	14.6	0.54	cbt	ind	15	oth	CDRS-C	2 (±1)	US	+	+	+	+	4
Topooco 2019	A	–	CO	17.5	0.96	cbt	Oth	8	oth	BDIII	2 (±1)	EU	+	+	sr	+	4
Vostanis 1996a	C	+	DD	12.7	0.56	cbt	ind	9	oth	MFQ C	2 (±1)	UK	–	–	+	–	1
Weisz 1997	C	–	CO	9.6	0.46	cbt	grp	8	cau	CDRS-R	2 (±1)	US	–	–	+	–	1
Wright 2020	A	+	CO	15.0	0.64	cbt	Oth	8	oth	BDI	2 (±1)	UK	+	+	sr	+	4
Young 2006	A	–	CO	13.4	0.85	ipt	Oth	10	oth	CESD	2 (±1)	US	+	–	+	+	3
Young 2016	A	–	CO	14.6	0.60	ipt	Oth	10	oth	CESD	2 (±1)	US	+	–	+	+	3
Yu 2002	C	–	CO	11.8	0.45	cbt	grp	10	cau	CDI	2 (±1)	Oth	–	–	sr	–	1

*A* adolescents, *A/C* adolescents or children, *A* allocation concealment, *Ba* blinded assessment, *Bat* behavioral activation therapy, *C* children, *Cau* care-as-usual, *Cbt* cognitive behavior therapy, *Clin* clinical versus other samples, *CO* scoring above a cut-off on a self-report instrument, *Cou* Country, *Ctr* type of control group, *DD* depressive disorder, *Diag* diagnosis, *EU* Europe, *Frm* format, *Grp* group, *Ind* individual, *Ipt* interpersonal psychotherapy, *Itt* intention-to-treat analyses, *Mage* mean age, *Nse* number of sessions, *Oth* Other, *Prop* *wom* proportion women, *Pst* problem-solving therapy, *Rob* risk of bias, *SD* subthreshold depression, *Sg* sequence generation, *Sr* self-report, *Sup* supportive therapy, *Type*: type of psychotherapy, *US* United States, *Wl* waiting list

Risk of bias was considerable. 21 of the 40 studies reported an adequate sequence generation (52.5%); 11 reported allocation to conditions by an independent party (27.5%); 21 reported using blinded outcome assessors (52.5%) while 14 used only self-report outcomes (35.0%). In 25 studies, intent-to-treat analyses were conducted (62.5%). Eight studies (20.0%) met all quality criteria, 19 studies (47.5%) met 2 or 3 criteria, and 13 met no or only one criterion (32.5%).

### Response rates in psychotherapy and control conditions at 2 (±1) months after baseline

The response rate (50% symptom reduction from baseline to post-treatment) at 2 (±1) months after baseline was available for 38 psychotherapy conditions and resulted in an overall response rate of 0.39 (95% CI: 0.34–0.45) (Table 2). The response rate was somewhat higher when it was calculated only on study completers (0.43; 95% CI: 0.37–0.49), and still higher when all study drop-outs were considered responders (0.47; 95% CI: 0.40–0.53). Exclusion of outliers did not materially affect the response rate. However, the sample of studies with low risk of bias resulted in a considerably smaller response rate (0.28; 95% CI: 0.18–0.42), although the confidence interval was wide because of low power. Heterogeneity was high in all analyses, except when outliers were excluded. The prediction interval of the response rate ranged from 0.63 to 4.45.

**Table 2:** Response rates, relative risks (RRs) and numbers-needed-to-be-treated (NNT) of psychotherapies versus control groups

		Psychotherapy					Control groups												
	*N*	Rate	95% CI	*I*^2^	95% CI		*N*	Rate	95% CI	*I*^2^	95% CI		*N*	RR	95% CI	*I*^2^	95% CI	NNT	95% CI
All psychotherapies	38	0.39	0.34–0.45	75	66–82		32	0.24	0.19–0.28	59	40–72		38	1.67	1.42–1.96	17	0–45	6.2	4.3–9.9
Completers only	38	0.43	0.37–0.49	74	65–81		32	0.25	0.21–0.30	56	35–70		38	1.68	1.45–1.95	2	0–39	5.9	4.2–8.9
All drop-outs responders	38	0.47	0.40–0.53	78	70–84		32	0.30	0.25–0.36	66	51–77		38	1.54	1.36–1.75	0	0–37	6.2	4.4–9.3
Outliers excluded	32	0.40	0.36–0.45	49	22–66		30	0.24	0.21–0.29	42	10–63		37	1.72	1.47–2.01	4	0–32	5.8	4.1–8.9
Only low Risk of Bias	7	0.28	0.18–0.42	87	75–93		6	0.16	0.11–0.23	57	0–83		7	1.56	1.03–2.37	12	0–74	11.2	4.6–208.3
Adjusted for publication bias	38	0.39	0.34–0.45	75	66–82		42	0.29	0.23–0.34	69	58–78		50	1.43	1.17–1.75	33	4–53	8.0	4.6–20.3
Long-term outcomes																			
Longer therapies (4–6 months)	7	0.29	0.21–0.39	74	45–88		7	0.19	0.14–0.25	47	0–78		7	1.52	0.99–2.32	28	0–69	10.1	n.s.
Follow-up at 6–12 months	12	0.44	0.37–0.51	64	34–81		11	0.33	0.24–0.43	80	64–88		12	1.39	1.11–1.74	44	0–72	7.8	4.1–27.5
Follow-up at 13–24 months	9	0.39	0.29–0.49	85	74–92		8	0.38	0.24–0.54	91	85–95		9	1.02	0.84–1.24	30	0–67	131.6	n.s.
Reliable improvement	38	0.54	0.46–0.62	85	81–89		32	0.32	0.26–0.39	78	69–84		38	1.59	1.35–1.88	69	56–78	5.3	3.6–8.9
Reliable deterioration	38	0.06	0.05–0.08	0	0–35		32	0.13	0.11–0.16	14	0–44		38	0.40	0.28–0.57	0	0–0	12.8	10.7–17.9
Recovery	8	0.58	0.49–0.67	52	0–79		6	0.36	0.22–0.54	76	45–89		8	1.84	0.99–3.44	45	0–75	3.3	n.s.

^a^Response rates for waitlist were not available after 6 months follow-up. Only two studies reported outcomes at longer than 24 months follow-up

^b^At 3–6 months follow-up, we calculated separate response rates for CAU and waitlist, but we pooled the response rates for psychotherapies although these were compared to CAU and waitlist

The pooled response rate in the 32 control conditions was 0.24 (95% CI: 0.19–0.28). It was marginally higher in the completers’ samples (0.25; 95% CI: 0.21–0.30) and somewhat higher when all dropouts were considered responders (0.30; 95% CI: 0.25–0.36). Excluding outliers and adjusting for publication bias resulted again in very comparable rates and it was considerably lower in studies with low risk of bias (0.16; 95% CI: 0.11–0.23). Heterogeneity was moderate to high in all analyses, except after excluding outliers.

**Fig. 2:**
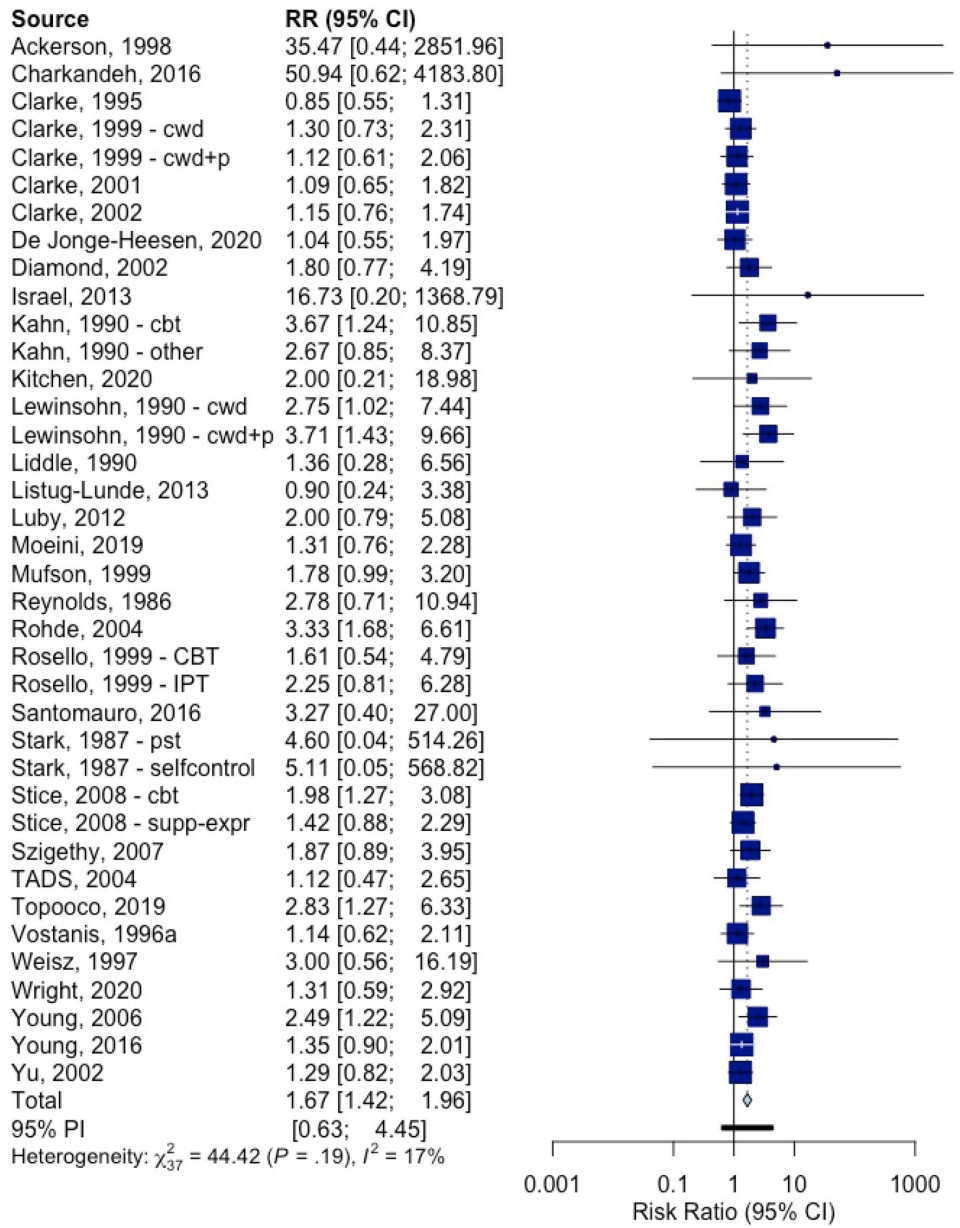
Forest plot: RR of response in psychotherapy versus control

**Fig. 3:**
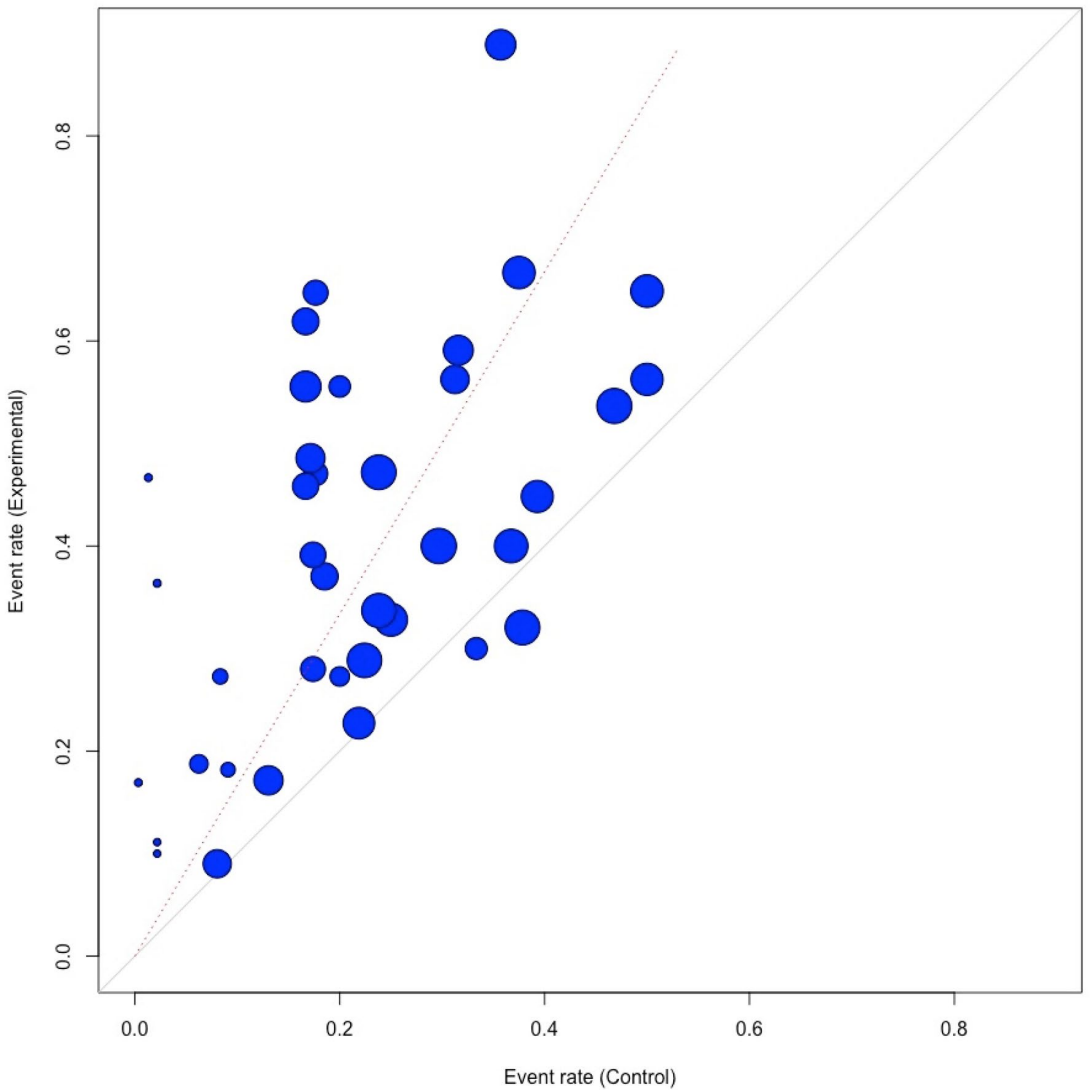
L’Abbé plot

The RRs of the therapy versus control conditions for the response rates and the NNTs are also reported in Table 2. The RR for response across all psychotherapies compared to control was 1.67 (95% CI: 1.42–1.96), and the NNT was 6.2 (95% CI: 4.3–9.9). The sensitivity analyses indicated broadly comparable outcomes, although the NNT was considerably larger in the studies with low risk of bias and to a lesser extent after adjustment for publication bias. The forest plot for the RR is presented in Fig. 2. The l’Abbé plot is given in Fig. 3.

### Other outcomes

The seven studies which lasted longer than 2 (±1) months but ended between 4 and 6 months after baseline, resulted in a lower response rate for the therapy conditions (0.29; 95% CI: 0.21–0.39) as well as for the control groups (0.19; 95% CI: 0.14–0.25). The relative risk and the NNT was not significant, possibly also because of the small number of studies.

Twelve studies that reported outcomes at 2 (±1) months follow-up also reported outcomes at 6–12 months and this reported in a response rate of 0.44 (95% CI: 0.37–0.51) for the therapy conditions, and 0.33 (95% CI: 0.24–0.43) for the control conditions. With a RR of 1.39 (95% CI: 1.11–1.74), the resulting NNT was 7.8. At 13–24 months follow-up, the response rates in therapy and control groups were very comparable, the RR was almost 1 and was not significant, nor was the NNT.

We were able to calculate the reliable change and deterioration in all 38 studies. The rate for reliable improvement was 0.54 (95% CI: 0.46–0.62) in the psychotherapy conditions and 0.32 (95% CI: 0.26–0.39) in the control conditions, with an RR of 1.59 (95% CI: 1.35–1.88) and a NNT of 5.3 (95% CI: 3.6–8.9). The deterioration rate was low in the therapy condition (0.06; 95% CI: 0.05–0.08) and was 0.13 in the control conditions (95% CI: 0.11–0.16). The RR was 0.40 (95% CI: 0.28–0.57) and the NNT was 12.8 (95% CI: 10.7–17.9), indicating that therapy reduced the chance of clinically significant deterioration.

Six studies (8 comparisons) included participants with a depressive disorder at baseline and reported the proportion no longer meeting these criteria. The proportion not meeting criteria at post-test was 0.58 (95% CI: 0.49–0.67) in the treatment group and 0.36 (95% CI: 0.22–0.54) in the control groups. The RR was 1.84 (95% CI: 0.99–3.44; n.s.) and the NNT was 3.3.

The subgroup analyses are reported in Table 3. No significant differences for the response rates within the psychotherapy conditions were found for any of the subgroup analyses. We did find significant differences in two subgroup analyses of the control conditions: one for diagnosis (the response rate was considerably higher in studies with youth meeting criteria for a depressive disorder or subthreshold depression, compared with studies in which participants had to score above a cut-off on a self-rating scale; *p*=0.04) and one for risk of bias (studies with low risk of bias resulted in a significantly lower response rate for control groups compared with other studies; *p*=0.01). For the RRs of the response rate versus control conditions, the only significant difference was found for type of control group (*p*=0.02).

**Table 3:** Subgroup analyses^a^

		Psychotherapy					Control groups													
	*N*	Rate	95% CI	*I*^2^	95% CI		*N*	Rate	95% CI	*I*^2^	95% CI		*N*	RR	95% CI	*I*^2^	95% CI		NNT	95% CI
Adolescents	28	0.41	0.34–0.48	80	71–86		24	0.24	0.19–0.30	64	45–77		28	1.68	1.38–2.04	28	0–55		6.1	4.0–11.0
Children	10	0.35	0.26–0.45	44	0–73		8	0.22	0.15–0.31	35	0–71		10	1.65	1.18–2.32	0	0–51		7.0	3.4–25.3
Only clinical samples	6	0.31	0.17–0.50	81	60–91		6	0.19	0.08–0.38	70	30–87		6	1.93	0.68–5.49	0	0–74		5.7	n.s.
Other recruitment	32	0.41	0.35–0.47	73	62–81		26	0.24	0.20–0.29	58	35–73		32	1.65	1.40–1.94	22	0–50		6.4	4.4–10.4
Depressive disorder	18	0.43	0.33–0.54	80	69–87		14	0.23	0.15–0.33	72	52–84		18	1.87	1.44–2.43	23	0–56		5.0	3.0–9.9
Subthreshold depression	5	0.40	0.33–0.49	53	0–83		4	0.32	0.25–0.40	29	0–74		5	1.34	0.85–2.11	54	0–83		9.2	n.s
Cut-off	15	0.34	0.25–0.43	70	49–82		14	0.21	0.17–0.26	25	0–60		15	1.64	1.25–2.15	0	0–44		7.4	4.1–19.0
CBT	25	0.39	0.31–0.46	79	70–86		23	0.23	0.18–0.29	67	50–79		25	1.64	1.30–2.07	36	0–61		6.8	4.1–14.5
IPT	4	0.59	0.36–0.79	84	60–94		3	0.32	0.24–0.40	0	0–68		4	1.72	1.10–2.70	0	0–83		4.3	1.8–31.3
Other	9	0.35	0.28–0.42	18	0–60		6	0.23	0.16–0.31	0	0–73		9	1.72	1.29–2.28	0	0–16		6.0	3.4–15.0
Individual	10	0.37	0.23–0.53	84	72–91		9	0.21	0.11–0.35	71	42–85		10	1.83	1.20–2.79	0	0–49		5.7	2.7–23.8
Group	20	0.41	0.34–0.48	67	47–79		16	0.26	0.20–0.32	58	26–76		20	1.55	1.23–1.95	35	0–62		7.0	4.0–16.7
Guided self-help/other	8	0.41	0.29–0.54	79	59–89		7	0.22	0.16–0.30	42	0–76		8	1.81	1.21–2.71	2	0–68		5.6	2.7–21.6
Low	7	0.28	0.18–0.42	87	75–93		6	0.16	0.11–0.23	57	0–83		7	1.56	1.03–2.37	12	0–74		11.2	4.6–208.3
Other	31	0.43	0.37–0.49	67	52–78		26	0.27	0.22–0.32	48	18–67		31	1.70	1.41–2.05	20	0–49		5.3	3.5–9.0
Waiting list	16	0.42	0.33–0.53	65	41–80		11	0.20	0.12–0.31	36	0–68		16	2.21	1.60–3.04	0	0–48		4.1	2.5–8.3
Usual care	12	0.37	0.31–0.44	49	1–74		11	0.28	0.22–0.36	60	22–79		12	1.34	1.07–1.68	6	0–61		10.5	5.3–51.0
Other	10	0.40	0.27–0.56	89	82–93		10	0.22	0.16–0.29	68	39–84		10	1.66	1.23–2.23	29	0–66		6.5	3.7–19.8

^a^None of the subgroup analyses indicated significant differences for the subgroups of response rates of psychotherapies; for the control groups the subgroups for Diagnosis (*p*=0.04) and for Risk of Bias (*p*=0.01) were significant, but none of the other subgroups; for the RRs of the therapies versus control conditions only the type of control condition was significant (*p*=0.02).

## Discussion

We examined rates for response, reliable improvement, reliable deterioration and recovery for psychotherapies aimed at children and adolescents with depression. We found that on average 39% of children and adolescents respond after getting treatment (at 1(±2) months after randomisation), while 24% respond in control conditions. The RR of responding in psychotherapy versus control was 1.66. The corresponding NNT was 6.3, which roughly means that a total of six patients need to receive therapy to have one more positive outcome, compared to the control group. Sensitivity analyses broadly supported these findings, although the rates were somewhat higher depending on whether dropouts were considered responders or not. Response rates were considerably lower in studies with low risk of bias, both within the psychotherapy and the control conditions and the NNT was considerably higher in these studies (NNT=11.2). Heterogeneity was high in the meta-analyses of response rates within the psychotherapy conditions and within the control conditions, but excluding outliers resulted in a comparable response rate and low heterogeneity.

Overall, the response rates are moderate, with about 60% of those receiving therapy not responding within 2 months. In the control conditions, this was considerably lower, but the additional benefit of therapies above the control condition is still modest. This means that the majority of children and adolescents do not respond to the therapies tested in these studies to date, and a considerable number would also have responded without therapy. These findings make clear that new, more effective treatment are needed to further reduce the burden of depression in these age groups. Future research should also examine potential reasons why children and adolescents do not respond and whether for example enhancing treatment fidelity, optimizing delivery methods, combination treatments, personalised approaches or sequential treatments may increase response rates. It should be noted that it is also important that future studies not only report continuous outcomes, but also binary outcomes such as response and remission, because of the clinical relevance of such outcomes.

The outcomes for reliable change and recovery are somewhat better than those for response, but still almost half of those receiving therapy do not reliably improve. We also found that the effects on response were retained at 6–12-month follow-up, which is encouraging because the effects do not disappear right after the end of therapy.

Effect sizes such as Cohen’s d and Hedges’ g are important indicators of the effectiveness of interventions, indicating the difference between treatment and control groups at post-test in terms of standard deviations. However, a disadvantage of effect sizes is that they do not indicate how many patients get better after treatment and how many in control conditions, although this is exactly the information that patients, parents, and clinicians want to know. This meta-analysis did present such numbers, which made it clear that many children and adolescents do not respond to treatment and that a considerable proportion respond in control groups.

One of the strong points of this study was that we could estimate clinically significant deterioration with the same method across all included studies. To the best of our knowledge, no previous meta-analysis has estimated negative effects of treatments for depression in youth. We found that 6% of youth receiving psychotherapy deteriorated, which was significantly lower than the 13% in the control conditions (NNT=5.1). It is encouraging that deterioration rates are lower in treatment than in control conditions, but 6% is still a large proportion. It is important that clinicians are aware of the fact that a considerable number of children and adolescents deteriorate while receiving treatment, and that strategies should be developed to handle deterioration.

In another study, we examined response rates in psychotherapies for adult depression [30]. We found an overall response rate of psychotherapies of 0.41 at 2 (±1) months follow-up, which is very comparable to the response rate of 0.39 found in the current study. The response rate in the control conditions was somewhat lower in the studies among adults (0.17) than in the studies in children and adolescents (0.24). This suggests that more children and adolescents get better in the control conditions than adults. This could explain that the effects of psychotherapies for depression are smaller in children and adolescents than in adults [8]. These findings have to be considered with caution, however, because of the high heterogeneity of these findings.

This study has several important limitations that should be taken into account when interpreting the effects. The most important limitation is that heterogeneity was very high, especially when estimating the response rates (less so for the RRs). This may be related to characteristics of the included studies that we did not examine in subgroup and metaregression analyses, such as treatment provider and proportion of participants using antidepressants. A complete review of all relevant characteristics is also beyond the scope of this study. Furthermore, such characteristics are often not consistently reported in the papers and reporting on the subsets of studies with clear characteristics could have produced an incomplete and perhaps invalid picture. However, the estimated rates appeared to be relatively robust and resulted in very comparable outcomes, in a series of sensitivity analyses. We also think, as we explained in the introduction that the clinical relevance of these outcomes is substantial and that pooling them is still important, as is also done for example in meta-analyses to estimate the prevalence of mental disorders [14–16]. Second, the response rates and the rates for clinically significant improvement and deterioration were based on estimates, using means, standard deviations and N at baseline and post-test. Although this method has been validated and correlates very highly with reported response rates, these are still estimates that may not reflect the actual response rates. “Individual patient data” meta-analyses could have calculated response rates directly. Third, response as outcome has been criticized, because it is depending on the baseline severity score, which may be unreliable [31]. Because other outcomes, such as remission cannot be standardized across different outcome measures, we do think that despite its weaknesses, response is the best measure to make a preliminary estimate of binary outcomes of treatment. Fourth, risk of bias was considerable in the large majority of trials. It was also notable that the studies with low risk of bias resulted in a considerably larger NNT.

Despite the limitations, this study showed that psychotherapies for depression in children and adolescents are effective compared to control conditions, but that still more than half of patients receiving therapy do not respond. Furthermore, a considerable number of those in control groups also respond. More effective treatments and treatments for those not responding to a first treatment are clearly needed.

## Data Availability

The data will be made available at the website of the meta-analysis project: www.metapsy.org

## Appendix 1

References of included studies

**Table:**

Study	Full references
Ackerson 1998	Ackerson, J., Scogin, F., McKendree-Smith, N., and Lyman, R. D. (1998). Cognitive bibliotherapy for mild and moderate adolescent depressive symptomatology. J Consult Clin Psychol, 66(4), 685-690.
Bolton 2007	Bolton, P., Bass, J., Betancourt, T., Speelman, L., Onyango, G., Clougherty, K. F., . . . Verdeli, H. (2007). Interventions for depression symptoms among adolescent survivors of war and displacement in northern Uganda: a randomized controlled trial [Randomized Controlled Trial; Research Support, Non-U.S. Gov't]. Jama, 298(5), 519-527. 10.1001/jama.298.5.519. (Accession No. CN-00590482)
Charkhandeh 2016	Charkhandeh, M., Talib, M. A., and Hunt, C. J. (2016). The clinical effectiveness of cognitive behavior therapy and an alternative medicine approach in reducing symptoms of depression in adolescents. Psychiatry research, 239, 325-330.
Clarke 1995	Clarke, G. N., Hawkins, W., Murphy, M., Sheeber, L. B., Lewinsohn, P. M., and Seeley, J. R. (1995). Targeted prevention of unipolar depressive disorder in an at-risk sample of high school adolescents: A randomized trial of a group cognitive intervention. Journal of the American Academy of Child & Adolescent Psychiatry, 34(3), 312-321.
Clarke 1999	Clarke, G. N., Rohde, P., Lewinsohn, P. M., Hops, H., and Seeley, J. R. (1999). Cognitive-behavioral treatment of adolescent depression: efficacy of acute group treatment and booster sessions. Journal of the American Academy of Child and Adolescent Psychiatry, 38(3), 272-279.
Clarke 2001	Clarke, G. N., Hornbrook, M., Lynch, F., Polen, M., Gale, J., Beardslee, W., Seeley, J. (2001). A randomized trial of a group cognitive intervention for preventing depression in adolescent offspring of depressed parents. Archives of general psychiatry, 58(12), 1127-1134.
Clarke 2002	Clarke, G. N., Hornbrook, M., Lynch, F., Polen, M., Gale, J., O’CONNOR, E., . . . Debar, L. (2002). Group cognitive-behavioral treatment for depressed adolescent offspring of depressed parents in a health maintenance organization. Journal of the American Academy of Child and Adolescent Psychiatry, 41(3), 305-313.
De Cuyper 2004	De Cuyper, S., Timbremont, B., Braet, C., De Backer, V., and Wullaert, T. (2004). Treating depressive symptoms in schoolchildren. European child and adolescent psychiatry, 13(2), 105-114.
De Jonge-Heesen 2020	De Jonge-Heesen KWJ, Rasing SPA, Vermulst AA, et al. Randomized control trial testing the effectiveness of implemented depression prevention in high-risk adolescents. BMC Medicine. 2020;18(1).
Diamond 2002	Diamond, G. S., Reis, B. F., Diamond, G. M., Siqueland, L., and Isaacs, L. (2002). Attachment-based family therapy for depressed adolescents: a treatment development study. Journal of the American Academy of Child and Adolescent Psychiatry, 41(10), 1190-1196.
Esposito‐Smythers 2019	Esposito‐Smythers, C., Wolff, J. C., Liu, R. T., Hunt, J. I., Adams, L., Kim, K., . . . Spirito, A. (2019). Family‐focused cognitive behavioral treatment for depressed adolescents in suicidal crisis with co‐occurring risk factors: a randomized trial. Journal of Child Psychology and Psychiatry, 60(10), 1133-1141. 10.1111/jcpp.13095
Gillham 2006	Gillham, J. E., Hamilton, J., Freres, D. R., Patton, K., and Gallop, R. (2006). Preventing depression among early adolescents in the primary care setting: A randomized controlled study of the Penn Resiliency Program. J Abnorm Child Psychol, 34(2), 195-211.
Idsoe 2019	Idsoe, T., Keles, S., Olseth, A. R., and Ogden, T. (2019). Cognitive behavioral treatment for depressed adolescents: Results from a cluster randomized controlled trial of a group course. BMC Psychiatry, 19(1). 10.1186/s12888-019-2134-3
Israel 2013	Israel, P., and Diamond, G. S. (2013). Feasibility of attachment based family therapy for depressed clinic-referred Norwegian adolescents. Clinical child psychology and psychiatry, 18(3), 334-350.
Kahn 1990	Kahn, J. S., Kehle, T. J., Jenson, W. R., and Clark, E. (1990). Comparison of cognitive-behavioral, relaxation, and self-modeling interventions for depression among middle-school students. School Psychology Review, 19(2), 196-211.
Kitchen 2020	Kitchen CEW, Tiffin PA, Lewis S, Gega L, Ekers D. Innovations in Practice: a randomised controlled feasibility trial of Behavioural Activation as a treatment for young people with depression. Child and Adolescent Mental Health. 2020.
Lewinsohn 1990	Lewinsohn, P. M., Clarke, G. N., Hops, H., and Andrews, J. (1990). Cognitive-behavioral treatment for depressed adolescents. Behavior Therapy, 21(4), 385-401.
Liddle 1990	Liddle, B., and Spence, S. H. (1990). Cognitive—Behaviour Therapy with Depressed Primary School Children: a Cautionary Note. Behavioural and Cognitive Psychotherapy, 18(2), 85-102.
Listug-Lunde 2013	Listug-Lunde, L., Vogeltanz-Holm, N., and Collins, J. (2013). A cognitive-behavioral treatment for depression in rural American Indian middle school students. American Indian and Alaska Native Mental Health Research: The Journal of the National Center, 20(1), 16-34.
Luby 2012	Luby, J., Lenze, S., and Tillman, R. (2012). A novel early intervention for preschool depression: findings from a pilot randomized controlled trial. Journal of Child Psychology and Psychiatry, 53(3), 313-322.
Martinović 2006	Martinović, Ž., Simonović, P., and Djokić, R. (2006). Preventing depression in adolescents with epilepsy. Epilepsy and behavior, 9(4), 619-624.
McCarty 2013	McCarty, C. A., Violette, H. D., Duong, M. T., Cruz, R. A., and McCauley, E. (2013). A randomized trial of the positive thoughts and action program for depression among early adolescents. Journal of Clinical Child and Adolescent Psychology, 42(4), 554-563.
Moeini 2019	Moeini, B., Bashirian, S., Soltanian, A. R., Ghaleiha, A., and Taheri, M. (2019). Examining the Effectiveness of a Web-Based Intervention for Depressive Symptoms in Female Adolescents: Applying Social Cognitive Theory. Journal of research in health sciences, 19(3), e00454-e00454.
Mufson 1999	Mufson, L., Weissman, M. M., Moreau, D., and Garfinkel, R. (1999). Efficacy of interpersonal psychotherapy for depressed adolescents. Archives of general psychiatry, 56(6), 573-579.
Mufson 2004	Mufson, L., Dorta, K. P., Wickramaratne, P., Nomura, Y., Olfson, M., and Weissman, M. M. (2004). A randomized effectiveness trial of interpersonal psychotherapy fordepressed adolescents. Archives of general psychiatry, 61(6), 577-584.
Reynolds 1986	Reynolds, W. M., and Coats, K. I. (1986). A comparison of cognitive-behavioral therapy and relaxation training for the treatment of depression in adolescents. Journal of Consulting and Clinical Psychology, 54(5), 653.
Rohde 2004	Rohde, P., Clarke, G. N., Mace, D. E., Jorgensen, J. S., and Seeley, J. R. (2004). An efficacy/effectiveness study of cognitive-behavioral treatment for adolescents with comorbid major depression and conduct disorder. Journal of the American Academy of Child and Adolescent Psychiatry, 43(6), 660-668.
Rosselló 1999	Rosselló, J., and Bernal, G. (1999). The efficacy of cognitive-behavioral and interpersonal treatments for depression in Puerto Rican adolescents. Journal of Consulting and Clinical Psychology, 67(5), 734.
Santomauro 2016	Santomauro, D., Sheffield, J., and Sofronoff, K. (2016). Depression in adolescents with ASD: a pilot RCT of a group intervention. Journal of autism and developmental disorders, 46(2), 572-588.
Stark 1987	Stark, K. D., Reynolds, W. M., and Kaslow, N. J. (1987). A comparison of the relative efficacy of self-control therapy and a behavioral problem-solving therapy for depression in children. J Abnorm Child Psychol, 15(1), 91-113.
Stice 2008	Stice, E., Rohde, P., Seeley, J. R., and Gau, J. M. (2008). Brief cognitive-behavioral depression prevention program for high-risk adolescents outperforms two alternative interventions: a randomized efficacy trial. Journal of Consulting and Clinical Psychology, 76(4), 595.
Szigethy 2007	Szigethy, E., Kenney, E., Carpenter, J., Hardy, D. M., Fairclough, D., Bousvaros, A., . . . Noll, R. (2007). Cognitive-behavioral therapy for adolescents with inflammatory bowel disease and subsyndromal depression. Journal of the American Academy of Child and Adolescent Psychiatry, 46(10), 1290-1298.
TADS 2004	Treatment for Adolescents With Depression Study (TADS) Team. Fluoxetine, Cognitive-Behavioral Therapy, and Their Combination for Adolescents With Depression Treatment for Adolescents With Depression Study (TADS) Randomized Controlled Trial. JAMA. 2004;292:807-820
Topooco 2019	Topooco, N., Byléhn, S., Dahlström Nysäter, E., Holmlund, J., Lindegaard, J., Johansson, S., . . . Andersson, G. (2019). Evaluating the Efficacy of Internet-Delivered Cognitive Behavioral Therapy Blended With Synchronous Chat Sessions to Treat Adolescent Depression: randomized Controlled Trial. Journal of medical Internet research, 21(11), e13393. 10.2196/13393
Vostanis 1996	Vostanis, P., Feehan, C., Grattan, E., and Bickerton, W.-L. (1996). Treatment for children and adolescents with depression: lessons from a controlled trial. Clinical child psychology and psychiatry, 1(2), 199-212.
Weisz 1997	Weisz JR, Thurber CA, Sweeney L, Proffitt VD, LeGagnoux GL (1997). Brief treatment of mild-to-moderate child depression using primary and secondary control enhancement training. Journal of Consulting and Clinical Psychology, 65, 703-707.
Young 2006	Young, J. F., Mufson, L., and Davies, M. (2006). Efficacy of interpersonal psychotherapy‐adolescent skills training: An indicated preventive intervention for depression. Journal of Child Psychology and Psychiatry, 47(12), 1254-1262.
Young 2016	Young, J. F., Benas, J. S., Schueler, C. M., Gallop, R., Gillham, J. E., and Mufson, L. (2016). A randomized depression prevention trial comparing interpersonal psychotherapy—Adolescent skills training to group counseling in schools. Prevention Science, 17(3), 314-324.
Yu 2002	Yu, D. L., and Seligman, M. E. (2002). Preventing depressive symptoms in Chinese children. Prevention and Treatment, 5(1), 9a.

## Code availability

The R syntax is available from the corresponding author.
